# Supplementary material for: Mental Toughness Development via Military-Style Training in the NCAA: A Three-Phase, Mixed-Method Study of the Perspectives of Strength and Conditioning Coaches
Source: Sports (Basel). 2022 Jun 9;10(6):92. doi: 10.3390/sports10060092 (PMC9230102; doi:10.3390/sports10060092)
Supplement: Supplementary file 1 [file sports-10-00092-s001.zip › sports-1708355-supplementary.pdf]

Questionnaire for Study 1

Mental Toughness: Transferability from the Military to Sport

1. Are you currently coaching in the NCAA?

    a. If Yes: Go to next question

    b. If No: Go to end of survey

2. Do you visit any military facilities (e.g., Fort Hood) with your team?

    a. If no: Goes directly to question #3 (see below).

    b. If yes.

        i. Is this your initiative?

        ii. If yes, goes to next question.

        iii. If no:

            1. Do you agree? Either yes or no moves to next question.

    a. Which sport?

    b. Gender of student-athletes?

        i. Season of the year (choose as many as they apply)?

            1. Offseason

            2. Preseason

            3. Inseason

            4. Postseason

    a. Is this part of Psychological Skill Training?

        i. If no: Goes directly to Demographics (see below)

        ii. If yes

        iii. Do you think it works on a significant level (i.e., transfers to the actual sport)?

        iv. Y or N

v. Is Mental Toughness one of the psychological constructs you're trying to develop with this visit?

vi. If no: Goes directly to Demographics (see below)

vii. If yes, will you be willing to measure MT pre and post to see if this actually works?

3. Do you invite ex-military (e.g., Navy Seals) on campus to talk/do workshops to your team?

   a. If no: Goes directly to question #4 (see below).

   b. If yes.

      i. Is this your initiative?

      ii. If yes, goes to next question.

      iii. If no:

         1. Do you agree? Either yes or no moves to next question.

   a. Which sport?

   b. Gender of student-athletes?

      i. Season of the year (choose as many as they apply)?

         1. Offseason

         2. Preseason

         3. Inseason

         4. Postseason

   a. Is this part of Psychological Skill Training?

      i. If no: Goes directly to Demographics (see below)

      ii. If yes

      iii. Do you think it works on a significant level (i.e., transfers to the actual sport)?

      iv. Y or N

v. Is Mental Toughness one of the psychological constructs you're trying to develop with this talk/workshop?

vi. If no: Goes directly to Demographics (see below)

vii. If yes, will you be willing to measure MT pre and post to see if this actually works?

4. Do you incorporate something similar in your training that involves any kind of military-style training?

a. If no: Goes directly to question #5 (see below).

b. If yes:

i. What is that (Open-ended question. Expand as much as you need)?

ii. Is this your initiative?

iii. If yes, goes to next question

iv. If no, do you agree? Either yes or no moves to next question.

1. Which sport?

2. Gender of student-athletes?

a. Season of the year (choose as many as they apply)?

i. Offseason

ii. Preseason

iii. Inseason

iv. Postseason

1. Is this part of Psychological Skill Training?

a. If no: Goes directly to Demographics (see below)

b. If yes

i. Do you think it works on a significant level (i.e., transfers to the actual sport)?

ii. Y or N

iii. Is Mental Toughness one of the psychological constructs you're trying to develop with this visit?

iv. If no: Goes directly to Demographics (see below)

v. If yes, will you be willing to measure MT pre and post to see if this actually works?

vi. Is Mental Toughness one of the psychological constructs you're trying to develop with this activity/training?

vii. If no: Goes directly to Demographics (see below)

viii. If yes, will you be willing to measure MT pre and post to see if this actually works?

5. Demographics

a. Gender

   i. F or M

b. Age

   i. In years (number)

c. Race

   i. White or Caucasian

   ii. Hispanic or Latino

   iii. Asian-Pacific Islander

   iv. Black or African-American

   v. Other

d. SCCC or MSCC?

e. Education

   i. Bachelor's

   ii. Master's

   iii. PhD

f. Other certifications (list as many as you have)

g. NCAA Division

   i. I

   ii. II

iii. III

h. Military background?

i. Y or N

Questionnaire for Study 2

Q1

Are you currently coaching in the NCAA?

Yes/No

Q2

Two years ago, triggered by incidents in Big 12, Pac-12, and Big Ten, we investigated military-style training in the strength and condoning field. When you were asked if you incorporate something in your training that involves any kind of military-style training, 22% (92 out of 421) of you said yes.

In more detail, you operationally defined it as a kind of training that entails: (1) Leadership Development (e.g., challenging team leaders to communicate clearly and with authority), (2) Mental Development (e.g., helping athletes concentrate while fatigued), (3) Physical Development (e.g., high intensity interval training), and (4) Team Development (e.g., team building calisthenics – on command as a team).

Q2a:

Do these themes match your understanding of how strength and conditioning coaches tend to use military style training with their athletes?

Yes/No

Q2b:

If not, please explain (e.g., Which theme is missing?)

Q3

The response rate mentioned above (22%) provides a snapshot that military-style training does not appear popular amongst CSCCa members that work in NCAA. Further, how CSCCa members operationally define military-style training may be different than how the media and general public understand military-style training. With that information in mind, what do you think most contributed to the media backlash the profession suffered?

Q4

What do you think CSCCa members can do to prevent similar media backlash in the future?

Q5

Demographics

a. Gender

i. F or M

b. Age

i. In years (number)

c. Race

i. White or Caucasian

ii. Hispanic or Latino

iii. Asian-Pacific Islander

iv. Black or African-American

v. Other

d. Education

i. Bachelor's

ii. Master's

iii. PhD

e. Other certifications (list as many as you have)

f. NCAA Division

i. I

ii. II

iii. III

g. Military background?

i. Y or N

Questionnaire for Study 3

Are you currently coaching in the NCAA?

Yes/No

First Wave

Three years ago, triggered by incidents in Big 12, Pac-12, and Big Ten, we investigated your perceptions on military-style training in the NCAA strength and condoning field.

We asked you a) if you visit any military facilities with your team; b) if you invite ex-military on campus to talk/do workshops with your team; and c) if you incorporate something similar in your training that involves any kind of military-style training?

Second Wave

A year ago, we tried to dig a bit deeper. So, we came back with some additional questions based on your first-wave answers.

We asked you a) if the operational definition of military-style training given by your peers matches your understanding of how strength and conditioning coaches tend to use military-style training with their athletes; b) what you think most contributed to the media backlash the profession suffered; and c) what you think CSCCa members can do to prevent similar media backlash in the future.

Third Wave

This year, we are concluding this long-lasting project by asking you five final emerging questions. Below, before each question, we are offering a summary of your relevant answers from the two previous waves.

Q1

When you are visiting military facilities with your team or inviting ex-military for workshops for military-style training, it is not your initiative.

Whose initiative is it to visit military facilities and/or invite ex-military on campus for workshops?

Q2

Football is the NCAA sport to which military-style training is most commonly applied.

Why is football the sport that you incorporate military-style training the most?

Q3

There is preliminary evidence that strength and conditioning coaches do not formally measure (e.g., via surveys) the effectiveness of their protocols on mental toughness levels. However, you perceive military-style training as an effective method of increasing mental toughness.

Without using a psychological questionnaire, how do you know that your military-style training increases your athletes' levels of mental toughness?

Q4

In the whole sample, military-style training is on average mostly used in men's teams. However, in volley-ball only, the results indicate the exact opposite.

Why do you use military-style training more often in women's than in men's volleyball teams?

Q5

There are empirically-based psychological interventions to increase mental toughness. Strength and conditioning coaches use physical interventions only.

Why don't you (also) use psychological interventions to increase your athletes' mental toughness?

Demographics

a. Gender

i. F or M

b. Age

i. In years (number)

c. Race

i. White or Caucasian

ii. Hispanic or Latino

iii. Asian-Pacific Islander

iv. Black or African-American

v. Other

e. Education

i. Bachelor’s

ii. Master’s

iii. PhD

f. Other certifications (list as many as you have)

g. NCAA Division

i. I

ii. II

iii. III

h. Military background?

i. Y or N
